# Looking for Novelty in an “Old” Receptor: Recent Advances Toward Our Understanding of GABA_A_Rs and Their Implications in Receptor Pharmacology

**DOI:** 10.3389/fnins.2020.616298

**Published:** 2021-01-14

**Authors:** David Castellano, Ryan David Shepard, Wei Lu

**Affiliations:** Synapse and Neural Circuit Research Section, National Institute of Neurological Disorders and Stroke, National Institutes of Health, Bethesda, MD, United States

**Keywords:** GABA, GABA_A_R, benzodiazepines, neurosteroids, pharmacology, LH4, Clptm1, Shisa7

## Abstract

Diverse populations of GABA_A_ receptors (GABA_A_Rs) throughout the brain mediate fast inhibitory transmission and are modulated by various endogenous ligands and therapeutic drugs. Deficits in GABA_A_R signaling underlie the pathophysiology behind neurological and neuropsychiatric disorders such as epilepsy, anxiety, and depression. Pharmacological intervention for these disorders relies on several drug classes that target GABA_A_Rs, such as benzodiazepines and more recently neurosteroids. It has been widely demonstrated that subunit composition and receptor stoichiometry impact the biophysical and pharmacological properties of GABA_A_Rs. However, current GABA_A_R-targeting drugs have limited subunit selectivity and produce their therapeutic effects concomitantly with undesired side effects. Therefore, there is still a need to develop more selective GABA_A_R pharmaceuticals, as well as evaluate the potential for developing next-generation drugs that can target accessory proteins associated with native GABA_A_Rs. In this review, we briefly discuss the effects of benzodiazepines and neurosteroids on GABA_A_Rs, their use as therapeutics, and some of the pitfalls associated with their adverse side effects. We also discuss recent advances toward understanding the structure, function, and pharmacology of GABA_A_Rs with a focus on benzodiazepines and neurosteroids, as well as newly identified transmembrane proteins that modulate GABA_A_Rs.

## Introduction

Efforts aimed at uncovering mechanisms driving inhibitory transmission have not only contributed to our understanding of nervous system function, but have also led to the development of several drugs used in the treatment of neurological and psychiatric disorders. In the brain, fast inhibitory transmission is predominantly mediated by GABA_A_ receptors (GABA_A_Rs), which are pentameric ligand-gated ion channels that conduct Cl^–^ upon activation (Olsen and Sieghart, 2009; Sigel and Steinmann, 2012; Gielen and Corringer, 2018). Thus far, nineteen GABA_A_R subunits, α(1–6), β(1–3), γ(1–3), δ, ρ(1–3), ε, θ, and π, have been identified in humans (Farrant and Nusser, 2005; Olsen and Sieghart, 2009; Sieghart and Savić, 2018) and the subunit composition, as well as arrangement, of GABA_A_Rs contribute to receptor properties such as trafficking, localization, kinetics, and pharmacology (Levitan et al., 1988a; Sigel et al., 1990; Lavoie et al., 1997; Belelli and Lambert, 2005; Herd et al., 2007; Sigel and Steinmann, 2012). Neuronal activity is dynamically regulated by both phasic and tonic inhibition resulting from GABA_A_Rs localized at synaptic or extrasynaptic regions, respectively (Mody and Pearce, 2004; Farrant and Nusser, 2005; Jacob et al., 2008; Lorenz-Guertin and Jacob, 2018; Chiu et al., 2019; Tomita, 2019). Furthermore, GABA_A_Rs are ubiquitously expressed across the brain, albeit in a region-, circuit- and cell-specific manner (Sieghart and Sperk, 2002; Engin et al., 2018). Deficits in GABAergic signaling are associated with the pathophysiology behind several neurological and psychiatric conditions (Macdonald et al., 2004; Ramamoorthi and Lin, 2011; Ben-Ari et al., 2012; Brickley and Mody, 2012; Hines et al., 2012; MacKenzie and Maguire, 2013; Rudolph and Möhler, 2014; Lorenz-Guertin et al., 2018) and many treatment strategies employ GABA_A_R-targeting drugs. Several therapeutic drug classes, including barbiturates, benzodiazepines, general anesthetics, and neurosteroids, target GABA_A_Rs at distinct allosteric binding sites and are commonly used to treat these disorders (Olsen and Sieghart, 2009; Olsen, 2018). Although they are widely employed for their sedative-hypnotic, anxiolytic, anticonvulsant, and/or muscle relaxant properties (Sieghart and Savić, 2018), adverse consequences such as drug dependence and withdrawal set limitations to their long-term use (Lalive et al., 2011; Tan et al., 2011; Balon and Starcevic, 2020). Thus, elucidating the structural and functional properties of GABA_A_Rs, as well as developing more selective GABA_A_R-targeting drugs with less side effects remain of high importance in modern drug discovery.

Contemporary studies on GABA_A_Rs have provided potentially and exciting opportunities for the development of more selective and efficacious drugs that target these receptors. Recent breakthroughs include new structural insights into ligand-bound GABA_A_Rs (Laverty et al., 2017, 2019; Miller et al., 2017; Zhu et al., 2018; Masiulis et al., 2019; Kim et al., 2020), the properties of different GABA_A_R subunit variants (Benkwitz et al., 2004; Boileau et al., 2010; Rajgor et al., 2020), and the recent characterization of transmembrane interacting proteins that modulate GABA_A_R trafficking and function (Davenport et al., 2017; Martenson et al., 2017; Yamasaki et al., 2017; Ge et al., 2018; Han et al., 2019, 2020). Although only initially characterized, these new observations of GABA_A_R accessory proteins add another layer of complexity to GABA_A_R regulation that should importantly be considered in drug screens and future pharmacology studies (Han et al., 2020). Our review will briefly focus on the role of GABA_A_R dysfunction in epilepsy, anxiety, and postpartum depression (PPD) as GABA_A_R-based pharmacotherapy is primarily employed as a treatment strategy in these conditions. Due to their widespread use in these disorders, an update on GABA_A_R function and pharmacology with respect to benzodiazepines and neurosteroids will be given. Additionally, we discuss recent studies on GABA_A_R structures, GABA_A_R subunit variants, and the discovery of GABA_A_R-associated transmembrane proteins. Lastly, we highlight potential opportunities in GABA_A_R pharmacology development as a result of these advancements.

## A Brief History on GABA_A_Rs as Prolific Drug Targets and Their Therapeutic Usage

**FIGURE 1:**
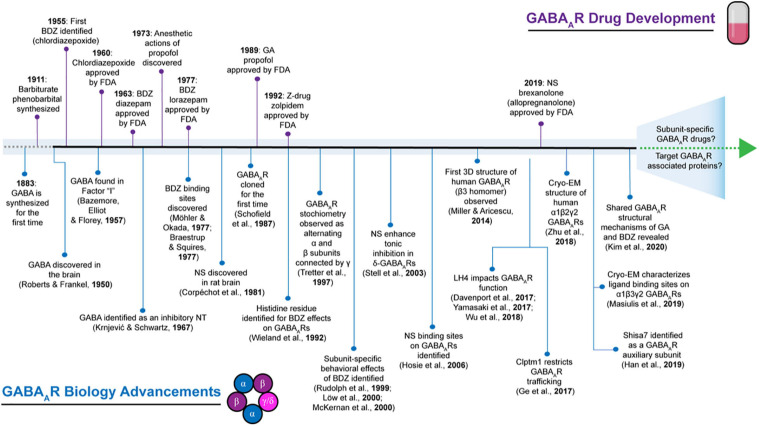
Historical timeline depicting select highlights that have advanced the field of GABA_A_R biology contrasted to events in GABA_A_R pharmaceutical development. BDZ, benzodiazepines; EM, electron microscopy; FDA: Food and Drug Administration; GA, general anesthetics; NS, neurosteroids; NT, neurotransmitter.

Drugs targeting GABA_A_Rs have been in use since the early 1900s (Figure 1), long before the isolation and cloning of receptor subunits in the 1980s (Sigel et al., 1982, 1983; Barnard et al., 1987; Schofield et al., 1987; Seeburg et al., 1990). Barbiturates were first employed for their anticonvulsant and sedative-hypnotic properties (Smart and Stephenson, 2019). However, a decline in their clinical use resulted from high mortality risk due to accidental overdose and the advent of benzodiazepines (López-Muñoz et al., 2005). The initial discovery of chlordiazepoxide in 1955 by Leo Sternbach at Hoffman-La Roche and diazepam (DZ) shortly after in 1959 created excitement for benzodiazepines (Figure 1), allowing them to become one of the most widely marketed and prescribed drugs (Mehdi, 2012). However, it was not accepted until decades later that adverse side effects such as addiction could occur with long-term usage (Lalive et al., 2011; Mehdi, 2012; Votaw et al., 2019). Although their site-of-action had not yet been determined, by the mid-to-late 1970s, it was known that barbiturates and benzodiazepines enhanced inhibition by potentiating the actions of GABA (Smart and Stephenson, 2019). Following the discovery of the various GABA_A_R subunits, many studies have been devoted toward characterizing the physiological and pharmacological properties of GABA_A_Rs with respect to subunit composition (Barnard et al., 1987; Schofield et al., 1987; Levitan et al., 1988b; Pritchett et al., 1989; Seeburg et al., 1990; Farrant and Nusser, 2005; Vithlani et al., 2011; Engin et al., 2018; Sieghart and Savić, 2018). GABA_A_Rs harbor several binding sites for barbiturates, benzodiazepines, general anesthetics, alcohol, and neurosteroids (Sieghart, 2015). Depending on the GABA_A_R subtype, many of these compounds exhibit differences in ligand sensitivity, resulting in different physiological and behavioral responses (Olsen and Sieghart, 2009). Accordingly, substantial work has been devoted toward understanding the binding mode and functional responses of various ligands at different GABA_A_R subtypes.

Given their pivotal role in mediating fast inhibitory neurotransmission, it is not surprising that alterations to GABA_A_R function are involved in many neurological and psychiatric disorders. GABA_A_R dysfunction has been observed in a myriad of conditions including seizures, sleep disorders, and anxiety-like disorders (Greenfield, 2013; Nuss, 2015; Engin et al., 2018; Amengual-Gual et al., 2019). Mutations in discrete GABA_A_R subunits have been shown to impair receptor properties, such as trafficking and ligand sensitivity (Hernandez and Macdonald, 2019; Maljevic et al., 2019). For instance, the first GABA_A_R subunit mutations associated with epilepsy were discovered in the γ2 subunit (Baulac et al., 2001; Wallace et al., 2001) and since then, a multitude of other mutations have been identified (Hernandez and Macdonald, 2019; Maljevic et al., 2019). Importantly, benzodiazepines remain as frontline drugs in attenuating seizures (Greenfield, 2013; Amengual-Gual et al., 2019) even though a variety of GABA_A_R-independent pathophysiological alterations can contribute to seizure generation (Stafstrom, 2010). Additionally, anxiety-like behaviors have also been observed concomitantly with dysregulation of inhibitory circuits (Smith and Rudolph, 2012; Smith et al., 2012; Nuss, 2015; Engin et al., 2018) and benzodiazepines continue to stand the test of time as effective anxiolytics (Balon and Starcevic, 2020). Apart from benzodiazepines, the therapeutic potential of neurosteroids have also garnered interest following the recent FDA approval of brexanolone (i.e., allopregnanolone; Mody, 2019) for postpartum depression (PPD) (Figure 1). During pregnancy, increased hormone production has been observed along with enhanced neurosteroid levels, such as allopregnanolone, and transient changes in allopregnanolone concentrations are implicated as a contributing factor to temporary changes in GABA-mediated inhibition, which can result in PPD-associated affective behaviors (Frye et al., 2011; Schüle et al., 2014). The therapeutic utility of neurosteroids in other forms of depression, anxiety, and epilepsies are also currently being explored (Lévesque et al., 2017; Czyk, 2019; Walton and Maguire, 2019; Zorumski et al., 2019; Belelli et al., 2020). We acknowledge that a variety of neurological and neuropsychiatric conditions involve GABA dysregulation (Ramamoorthi and Lin, 2011; Mele et al., 2019), but we have chosen to focus on disorders where GABA_A_R pharmacology is currently and commonly used: benzodiazepines for epilepsy and anxiety, as well as neurosteroids for PPD. Individuals suffering from these conditions stand the most to gain from the development of novel therapeutic GABA_A_R-targeting drugs that achieve higher specificity and efficacy, while also mitigating adverse side effects.

## Benzodiazepine Actions on GABA_A_Rs

GABA_A_Rs with high sensitivity to benzodiazepines are typically tri-heteromeric and composed of two α 1-3, 5, two β (1-3), and one γ2 subunit (Farrant and Nusser, 2005). Similar to GABA, subunit composition alters sensitivity to benzodiazepines (Minier and Sigel, 2004). The classical, high-affinity benzodiazepine binding site is present at the extracellular α-γ interface and upon binding, benzodiazepines can increase GABA affinity (Twyman et al., 1989; Lavoie and Twyman, 1996), modulate gating by priming the receptor toward a preactivated step, and affect the rate of desensitization (Twyman et al., 1989; Gielen et al., 2012; Goldschen-Ohm et al., 2014; Jatczak-Śliwa et al., 2018). With respect to single-channel properties, benzodiazepines increase the frequency of channel opening and bursting, with no effect on conductance or channel opening duration (Twyman et al., 1989; Rogers et al., 1994). The actions of benzodiazepines observed at the microscopic level continue to shape our understanding of how inhibitory postsynaptic currents (IPSCs) are modulated. Accordingly, in the presence of benzodiazepines, IPSC decay is prolonged and at some synapses, an increase in amplitude has also been observed (Möhler et al., 2002; Mozrzymas et al., 2007; Karayannis et al., 2010). The efficacy of benzodiazepine potentiation ranges from partial to full allosteric modulation, which is dependent on the binding mode of the benzodiazepine ligand and its effects on GABA_A_R gating properties (Elgarf, 2018).

Given that the high affinity binding site exists at the α–γ interface, a multitude of side effects can occur following administration due to their non-selective targeting of GABA_A_Rs. Classical benzodiazepines interact non-selectively with all GABA_A_Rs containing α1-, α2-, α3-, α5-, and γ2 subunits (Moody and Jenkins, 2018). Whereas α1-containing GABA_A_Rs have the greatest distribution throughout the brain (Möhler et al., 2002), α2-, α3-, and α5-containing GABA_A_Rs are expressed with greater confinement to specific brain regions (Engin et al., 2018). Modulation of α1- and α2-containing GABA_A_Rs are associated with the sedative (Rudolph et al., 1999; McKernan et al., 2000; Rowlett et al., 2005) and anxiolytic (Löw et al., 2000) properties of benzodiazepines, respectively. In addition, α3-containing GABA_A_Rs have also been implicated with the anxiolytic effects of benzodiazepines (Atack et al., 2005; Dias et al., 2005), but this has also been disputed (Löw et al., 2000; Behlke et al., 2016). Lastly, α5-containing GABA_A_Rs may also mediate certain aspects of anxiety behaviors (Botta et al., 2015) and modulation of this subtype can result in anxiolysis (Behlke et al., 2016). As a result of the predominant behavioral effects produced by α1- and α2-containing GABA_A_Rs, there have been attempts to develop novel compounds that target these GABA_A_R subtypes specifically. Most notably, non-benzodiazepines (also known as z-drugs) were developed and are used as sleep aids due to their higher selectivity for α1-containing GABA_A_Rs (Atack, 2011; Tan et al., 2011; Cheng et al., 2018; Sieghart and Savić, 2018). However, there are currently no FDA-approved, subtype-selective GABA_A_R-targeting drugs that function as anxiolytics and are devoid of sedative properties.

One concern associated with long-term usage of benzodiazepines is the development of tolerance based on observations of reduced GABAergic transmission along with altered subunit composition following chronic benzodiazepine treatment (Uusi-Oukari and Korpi, 2010; Jacob et al., 2012; Vinkers and Olivier, 2012; Lorenz-Guertin et al., 2019). The development of benzodiazepine tolerance likely results from decreased GABA_A_R surface availability due to enhanced inhibition from prolonged drug exposure (Gallager et al., 1984). Interestingly, changes in GABA_A_R expression appear to be subunit-specific (Uusi-Oukari and Korpi, 2010) and are also dependent on cell-type and brain region (Poisbeau et al., 1997; Jacob et al., 2012; Foitzick et al., 2020). For example, prolonged DZ treatment has been observed to reduce total γ2 expression due to increased lysosomal degradation of this subunit (Lorenz-Guertin et al., 2019). Phosphorylation of GABA_A_Rs by specific protein kinase C (PKC) isoforms have been demonstrated to impact benzodiazepine sensitivity in recombinant GABA_A_Rs (Leidenheimer et al., 1992; Qi et al., 2007; Nakamura et al., 2015). In addition, calcineurin-dependent dephosphorylation following application of DZ has also been shown to induce endocytosis of GABA_A_Rs (Nicholson et al., 2018). Although benzodiazepines non-selectively target α1-3,5/γ2-containing GABA_A_Rs throughout the brain, it is possible that variability in benzodiazepine sensitivity among different cell types arises from changes in GABA_A_R subunit post-translational modifications (PTMs) and/or their interactions with GABA_A_R-interacting proteins (Jacob et al., 2012). Together, these features could result in variable GABA_A_R turnover rate in different neuronal populations and may explain why benzodiazepine tolerance for the sedative-hypnotic effects occur more rapidly in comparison to the anticonvulsant/anxiolytic actions (Bateson, 2002; Vinkers and Olivier, 2012), but this supposition requires further clarification.

Furthermore, although acute treatment with benzodiazepines is generally considered safe, their chronic use can result in physical dependence (Soyka, 2017; Silberman et al., 2020) and potentially drug abuse/misuse (Lalive et al., 2011; Tan et al., 2011), which is a major public health concern across the world (Votaw et al., 2019). In general, drugs of abuse “hijack” brain reward circuitry leading to enhanced dopamine (DA) release from the ventral tegmental area (VTA) into the nucleus accumbens (NAc) (Kauer and Malenka, 2007). Mechanistically, benzodiazepines activate α1-containing GABA_A_Rs on GABAergic interneurons in the VTA, resulting in disinhibition which promotes DA release (Tan et al., 2010). Additionally, activation of α2-containing GABA_A_Rs in the NAc have also been shown to mediate reward learning associated with benzodiazepines (Reynolds et al., 2012; Engin et al., 2014). It is important to note that the abuse liability for benzodiazepines is the highest in individuals who use other drugs of abuse (Silberman et al., 2020). Additionally, the development of physical dependence can occur independently of addiction (Silberman et al., 2020). Lastly, how tolerance develops requires further elucidation, due to observations that the development of tolerance differs based on the usage of the benzodiazepine (Vinkers and Olivier, 2012). One potential reason for disparities observed in tolerance and risk of misuse is due to the overall half-life of the type of benzodiazepine (Vinkers and Olivier, 2012). Thus, targeting GABA_A_Rs based on subunit-specificity continues to remain important not only toward understanding the roles of different subtypes in circuits and behaviors, but also for achieving optimal therapeutic efficacy while mitigating adverse effects.

## Neurosteroid Actions on GABA_A_Rs

In contrast to the historical usage of benzodiazepines as pharmacotherapy, neurosteroids are the newest class of GABA_A_R-targeting drugs, with brexanolone as the first therapy indicated for the treatment of PPD (Cristea and Naudet, 2019; Mody, 2019). Neurosteroids are robust modulators of both synaptic and extrasynaptic GABA_A_Rs (Belelli and Lambert, 2005; Belelli et al., 2020). Thus, their effects on GABAergic transmission are mediated by prolonging phasic inhibition, as well as increasing tonic conductance. Neurosteroid binding sites on GABA_A_Rs were initially discovered within the transmembrane domains of the α1β2γ2 between the α and β subunit interface. and key residues regulating their binding are largely conserved among α2-5/β3γ2 and α4β3δ subtypes (Hosie et al., 2006, 2009). Additionally, neurosteroids potentiate GABA-mediated currents with higher efficacy in δ-containing GABA_A_Rs compared to γ2-containing GABA_A_Rs (Bianchi and Macdonald, 2003; Belelli et al., 2020). Comparable to benzodiazepines, neurosteroids increase the frequency of single channel openings, but also prolong the channel open duration similarly to barbiturates (Twyman and Macdonald, 1992; Belelli et al., 2020). Furthermore, neurosteroids enhance the duration of IPSCs by prolonging the decay time (Lambert et al., 2003). Interestingly, relatively high concentrations (>100 nM) of neurosteroids can directly activate GABA_A_Rs in the absence of GABA (Belelli and Lambert, 2005). These observations may potentially be clinically relevant given that altered neurosteroid concentrations in the brain have been observed in neuropsychiatric conditions such as PPD, anxiety, and stress (Purdy et al., 1991; Schumacher et al., 2003; Belelli et al., 2020).

It has been previously demonstrated that knock-in mice with α2-containing GABA_A_Rs rendered insensitive to neurosteroid potentiation for this subtype exhibit anxiety-like behaviors without displaying depressive-like phenotypes nor effects on analgesia (Durkin et al., 2018). These observations potentially suggest a specific role for how neurosteroids might be useful as anxiolytics given that α2-containing GABA_A_Rs have already been associated with the anxiolytic effects of benzodiazepines (Löw et al., 2000). While the behavioral effects of neurosteroids can be interrogated based on their interaction with distinct GABA_A_R subtypes (Belelli et al., 2020), achieving subtype-selectivity may prove challenging due to conserved binding sites among synaptic and extrasynaptic GABA_A_Rs (Hosie et al., 2007, 2009). Neurosteroid modulation of GABA_A_Rs are also regulated by phosphorylation status, which affects receptor expression and/or surface trafficking, as well as neurosteroid sensitivity (Smith et al., 2007; Abramian et al., 2014; Comenencia-Ortiz et al., 2014; Nakamura et al., 2015). Distinct neurons have been observed to exhibit differences in their sensitivity to neurosteroids due to changes in phosphorylation mediated by different kinases, such as protein kinase A (PKA) or PKC (Fáncsik et al., 2000; Hodge et al., 2002; Vicini et al., 2002; Harney et al., 2003; Kia et al., 2011). Together, both phosphorylation of GABA_A_R subunits and changes to endogenous neurosteroid levels can have profound effects on phasic and tonic inhibition, contributing to the heterogeneity of inhibition across brain regions.

There has been growing interest in the therapeutic development of neurosteroids for potential application in other forms of depression such as major depressive disorder (zuranolone*; Sage Therapeutics*) and treatment-resistant depression (zuranolone*; Sage Therapeutics*, ganaxolone; *Marinus Pharmaceuticals*). Indeed, it has been observed that neurosteroid levels are reduced in depression and treatment with various antidepressants can normalize these concentrations (Lüscher and Möhler, 2019). Given the comorbidity of anxiety and depression, the potential for neurosteriods being employed as effective anxiolytics are also being explored (Schüle et al., 2014; Czyk, 2019; Gunduz-Bruce et al., 2019; Lüscher and Möhler, 2019; Zorumski et al., 2019). Additionally, neurosteroids are also being considered for use in seizure disorders such as refractory status epilepticus and PCDH19-related epilepsy (ganaxolone; *Marinus Pharmaceuticals*). Polypharmacy involving administration of both benzodiazepines and neurosteroids together are also being considered for mitigating epileptic seizures (Rogawski et al., 2020). Taken together, there is the possibility that further development of neurosteroids as a therapeutic agent will be applicable to other neurological disorders.

## Recent Advances in GABA_A_R Biology

Extensive characterization into the physiological and pharmacological properties of distinct GABA_A_Rs continues to provide insight toward their relevance at the cellular, circuit, and behavioral level. The past two decades have seen the development of subtype-selective compounds targeting GABA_A_Rs with specific pharmacological and behavioral actions (Rudolph and Knoflach, 2011; Sieghart and Savić, 2018; Chen et al., 2019). Additionally the recent emergence of structural data revealing GABA_A_Rs bound with different ligands will certainly help refine and guide drug development (Olsen et al., 2019). Differences in expression profiles and receptor function among GABA_A_R subtypes, subunit variants due to alternative splicing (Boileau et al., 2003, 2010; Eom et al., 2011; Miller et al., 2018), and mechanisms that preferentially enrich mRNA transcripts of discrete GABA_A_R subunits at specific subcellular regions (Rajgor et al., 2020), continue to highlight the diversity of GABAergic signaling in neuronal inhibition. Furthermore, the discovery of transmembrane proteins that associate with GABA_A_Rs are beginning to shed light on their molecular mechanisms *in vivo* and may also provide strategies for targeting select GABA_A_R subtypes and in turn, lead to the development of more effective therapeutics for neurological and psychiatric disorders.

### Structural Insights Into GABA_A_Rs and Signaling Mechanisms

Molecular modeling of drug-receptor interactions relies on high-resolution structures and aims to characterize not only the spatial architecture of the receptor, but how ligand binding induces changes in receptor conformation and influences channel gating (Olsen et al., 2019). Several structures of AMPA receptors (AMPARs) bound to ligands and/or in complex with auxiliary subunits help exemplify how these interactions are critical for modulating AMPAR function (Herguedas et al., 2019; Nakagawa, 2019; Zhao et al., 2019; Kamalova and Nakagawa, 2020). In contrast, until recently, there has been a lack of structural data regarding GABA_A_Rs, which has limited our understanding of how these receptors structurally interact with their ligands. New studies using cryogenic electron microscopy (cryo-EM) to examine full-length, synaptic GABA_A_Rs are now available and will be important for bridging the link between receptor architecture and their pharmacological signaling mechanisms (Laverty et al., 2019; Masiulis et al., 2019; Kim et al., 2020). In addition to high affinity benzodiazepine sites at the α-γ interface, other benzodiazepine binding sites on GABA_A_Rs have also been inferred (Walters et al., 2000; Baur et al., 2008; Ramerstorfer et al., 2011; Wongsamitkul et al., 2017; Sigel and Ernst, 2018; Lian et al., 2020). Recently, cryo-EM studies confirmed the presence of a low-affinity binding site present within the transmembrane domain of the αβ interface as described in earlier studies (Walters et al., 2000; Ramerstorfer et al., 2011; Sigel and Ernst, 2018; Masiulis et al., 2019; Lian et al., 2020). Moreover cryo-EM studies have identified the presence of other distinct benzodiazepine binding sites, specifically within the β-α and γ-β interfaces (Masiulis et al., 2019; Kim et al., 2020). It has been shown that DZ concentration-response curves exhibit biphasic responses (Walters et al., 2000; Baur et al., 2008; Ramerstorfer et al., 2011; Wongsamitkul et al., 2017). Potentiation of GABA by high concentrations of DZ (>20 μM) is thought to be mediated by a low-affinity binding site and submaximal GABA responses from α1β2 receptors lacking the γ2 subunit have been shown to be potentiated by high concentrations of DZ, which were not blocked by flumazenil (Walters et al., 2000; Baur et al., 2008; Ramerstorfer et al., 2011; Wongsamitkul et al., 2017). Although the amount of DZ used in this particular study far exceeds the concentration needed for their therapeutic effects (<0.3 μM), activation of this low-affinity site may potentially mediate the anesthetic properties of benzodiazepines (Walters et al., 2000; Olsen, 2018; Masiulis et al., 2019; Kim et al., 2020). While this study only assessed the effects of DZ in α1β2 and α1β2γ2 GABA_A_Rs (Walters et al., 2000), it should be noted that other benzodiazepine binding sites distinct from the α-γ interface appear to be present in cryo-EM structures of both α1β2γ2 (Kim et al., 2020) and α1β3γ2 GABA_A_Rs (Walters et al., 2000; Olsen, 2018; Masiulis et al., 2019). Interestingly, when comparing the binding of partial and full benzodiazepine agonists, partial benzodiazepine agonists appear to sit deeper within the α/γ binding pocket and could explain differences in efficacy between DZ and bretazenil, respectively (Masiulis et al., 2019). Although these studies highlight the discovery of other unique benzodiazepine binding sites, there are currently no studies that have demonstrated that these unique, low-affinity binding sites can mediate the anesthetic effects of benzodiazepines in isolation. In the future, it would be interesting to explore the conditions that facilitate the activity of GABA_A_Rs through these low-affinity sites and whether these sites can be selectively targeted. It also is possible that future cryo-EM structures may reveal subtle differences in the benzodiazepine binding pocket among distinct, benzodiazepine-sensitive GABA_A_Rs, which could help in the development of more subtype-selective compounds.

Structural data has also highlighted the importance of key residues (Hosie et al., 2006) and the binding modes of distinct neurosteroid ligands on GABA_A_Rs (Laverty et al., 2017; Miller et al., 2017; Sugasawa et al., 2020). Previously, it was thought that the allosteric modulation and direct activation of GABA_A_Rs by neurosteroids were due to binding on distinct sites, at the α subunit and the αβ interface, respectively (Hosie et al., 2006). However, structures of GABA_A_R chimeras bound with tetrahydrodeoxycorticosterone (THDOC) later revealed that both actions resulted from binding at the same site (Laverty et al., 2017). It was also shown that neurosteroids, such as pregnanolone sulfate, occupy a separate site within the intra-subunit transmembrane domain of the α subunit distinct from THDOC (Laverty et al., 2017). Accordingly, it has recently been shown that different neurosteroid ligands can promote either the activation or desensitization of α1β3 GABA_A_Rs by binding to the intersubunit interface of the β-α subunits or within the intrasubunit on β3, respectively (Laverty et al., 2017; Miller et al., 2017; Sugasawa et al., 2020). Currently, it remains unknown whether neurosteroid binding sites among synaptic and extrasynaptic GABA_A_R are structurally distinct when ligand bound and if these differences can be exploited for the development of novel neurosteroids with varying efficacy and specificity. However, to date, there are a lack of neurosteroid-based ligands designed for subtype-selectivity (Althaus et al., 2020), such as exclusively targeting α2-containing GABA_A_Rs for their anxiolytic properties without modulating α4-containing GABA_A_Rs. Similar to benzodiazepines, future cryo-EM studies may also uncover unique differences among neurosteroid binding sites in different GABA_A_R subtypes that can be therapeutically leveraged.

### Spatial Expression Profiles of GABA_A_R Subtypes and GABA_A_R Subunit Variants

The circuit and behavioral roles mediated by specific GABA_A_R subtypes are dependent on their abundance in precisely defined regions. High GABA_A_R sensitivity to benzodiazepines is conferred by a histidine residue in the N-terminal extracellular region (Wieland et al., 1992) and is specific to GABA_A_Rs containing α1-, α2-, α3-, and/or α5 subunits. However, GABA_A_Rs containing these subunits can be found across the brain and overall modulation of these GABA_A_Rs contributes to both the desired therapeutic effect, but also some of the side effects. Therefore, it is important to understand how brain regions are modulated in a circuit-specific manner and the physiological role of GABA_A_Rs subtypes within brain regions.

With respect to the anxiolytic effects of benzodiazepines, the amygdala has received prominent attention due to its role in mediating emotional responses and this structure can be divided into discrete subdivisions based on cell-type, circuit, and physiological role (Ehrlich et al., 2009). Differences in GABA_A_R subtypes expressed in subdivisions of the amygdala have been observed (Fritschy and Mohler, 1995; Pirker et al., 2000; Kaufmann et al., 2003; Fujimura et al., 2005). Although nearly all GABA_A_Rs are expressed throughout the amygdala, the region and cellular localization of these GABA_A_R subtypes can impact anxiety behaviors (Engin et al., 2018). For example, in the central amygdala (CeA), α5-containing GABA_A_Rs are associated with anxiety-like behaviors in a cell-specific manner (Haubensak et al., 2010; Botta et al., 2015). Specifically, knockdown (KD) of α5-containing GABA_A_Rs in PKCδ+ neurons results in anxiogenesis, highlighting the importance of subtype- and cell-specific regulation of anxiety-like behaviors (Haubensak et al., 2010; Herman et al., 2013; Botta et al., 2015). Additionally, α1-containing GABA_A_Rs are localized on corticosterone releasing factor neurons and have been shown to contribute to anxiety-like phenotypes possibly through the regulation of neuronal excitability (Gafford et al., 2012; Herman et al., 2013). However, the specific role of α1-containing GABA_A_R activity within the CeA with respect to control over anxiety-like behaviors has yet to fully be determined (Engin et al., 2018). In line with these observations, benzodiazepines have also been shown to impact CeA activity and anxiety-like behaviors (Carvalho et al., 2012; Botta et al., 2015; Griessner et al., 2018). Although this could also be due to a higher degree of α2-containing GABA_A_R expression (Fritschy and Mohler, 1995; Pirker et al., 2000), the cell-type and circuit-specific role of α2-containing GABA_A_Rs in CeA is not completely understood (Engin et al., 2018). Further complications teasing out the effects of benzodiazepines come from the fact that benzodiazepines non-selectively modulate all α1-3, 5-containing GABA_A_Rs distributed across the entire amygdala (Fritschy and Mohler, 1995; Pirker et al., 2000; Engin et al., 2018). Taken together, the complexity regarding the microcircuitry governing anxiety and how both region- and cell-specific expression of GABA_A_R subtypes can impact anxiety behaviors still requires further investigation.

Furthermore, subcellular localization of GABA_A_Rs also profoundly determines the type of neuronal inhibition exhibited and how GABA_A_R-targeting drugs will modulate neuronal activity (Kerti-Szigeti and Nusser, 2016; Nathanson et al., 2019; Kramer et al., 2020). The mobility and diffusion of GABA_A_Rs between synaptic and extrasynaptic regions are dependent on subunit composition, specific motifs within the intracellular domain of certain subunits, and the interaction of GABA_A_Rs with scaffolding partners such as gephyrin or radixin (Jacob et al., 2005; Thomas et al., 2005; Loebrich et al., 2006; Bannai et al., 2009; Mukherjee et al., 2011; Tyagarajan and Fritschy, 2014; Hausrat et al., 2015; Hannan et al., 2019; Davenport et al., 2020). Recently, there have been reports of differences in synaptic and extrasynaptic GABA_A_R subunit localization, as well as neuron-specific differences (Schulz et al., 2018; Magnin et al., 2019). For example, it is well-established that in hippocampal pyramidal neurons, the α5 subunit is predominantly expressed at extrasynaptic regions and mediates the majority of tonic inhibition (Crestani et al., 2002; Caraiscos et al., 2004; Farrant and Nusser, 2005; Glykys and Mody, 2007; Glykys et al., 2008). Interestingly, α5-containing GABA_A_Rs can also localize synaptically and contribute to phasic inhibition (Brady and Jacob, 2015; Schulz et al., 2018; Davenport et al., 2020). Furthermore, in hippocampal somatostatin interneurons, α5-containing GABA_A_Rs appear to be localized synaptically as they co-localize with VGAT and are targeted by vasoactive intestinal polypeptide (VIP)- and calretinin-expressing (Schulz et al., 2018; Magnin et al., 2019), but not parvalbumin interneurons. In addition, α5-GABA_A_Rs targeted by VIP interneurons appear to be involved in anxiety-like behaviors (Magnin et al., 2019). More work is needed to understand how distinct GABA_A_Rs are targeted by different inhibitory inputs and how differences in compartment localization can dynamically regulate neuronal activity. For example, it is currently unknown whether pharmacological targeting of perisomatic or dendritic inhibition exclusively can result in better therapeutic efficacy. Although currently not feasible, future endeavors examining the specific roles of GABA_A_Rs and their contributions to perisynaptic and/or dendritic inhibition will provide a more mechanistic understanding of input-specific inhibition and could perhaps allow these mechanisms to be manipulated pharmacologically.

In addition, GABA_A_R splice variants with differences in function and pharmacology continue to further enhance the diversity and classification of GABA_A_R subtypes (Whiting et al., 1990; Boileau et al., 2010; Miller et al., 2018; Smart and Stephenson, 2019; Rajgor et al., 2020). For example, the γ2 subunit can exist as either a long (γ2L) or short (γ2S) variant, with the latter missing eight amino acids in the long intracellular loop (Whiting et al., 1990; Boileau et al., 2010). Functionally, γ2S differs from γ2L in zinc sensitivity and kinetics, and the surface expression of γ2S alone is possible even when co-transfected with α and β subunits (Boileau et al., 2003, 2010). Additionally, γ2L and γ2S expression changes over the course of development and their expression is confined to different brain regions (Wang and Burt, 1991; Gutiérrez et al., 1996). Noteworthily, it has also been observed that in schizophrenia, γ2S is decreased (Huntsman et al., 1998) which elicits the question as to whether γ2L and/or γ2S are differentially involved in other neurological and neuropsychiatric conditions. Lastly, PTMs can influence GABA_A_R properties and phosphorylation of the serine site S343 which is exclusive to the γ2L variant and not in γ2S (Lorenz-Guertin et al., 2018) has been documented (Nakamura et al., 2015). However, more research is required in order to identify other residues within the γ2L and γ2S variants that are subject to phosphorylation or other PTMs, as well as how these PTMs modulate GABA_A_R properties. With newer tools that can achieve greater drug targeting specificity (Shields et al., 2017; Atasoy and Sternson, 2018; Rao et al., 2019; Crocetti and Guerrini, 2020) along with rational drug design (Antkowiak and Rammes, 2019; Scott and Aricescu, 2019), it may be possible in the near future for drug development strategies to exploit these differences in order to maximize the therapeutic efficacy of newer compounds while also decreasing the likelihood of unwanted side effects by “sparing” other GABA_A_R subtypes.

### GABA_A_R-Associated Transmembrane Proteins

Although a majority of compounds have been developed that target the pore-forming subunits of GABA_A_Rs, recent advances in the field of GABA_A_R biology have illuminated that native GABA_A_R exist as a complex with other accessory proteins rather than in isolation (Khayenko and Maric, 2019). Within the past decade (Figure 1), novel transmembrane proteins have been discovered that associate with native GABA_A_Rs and have distinct effects on receptor trafficking, kinetics, and/or pharmacology (see Han et al., 2020 for an in-depth review). Therefore, these proteins present themselves as potentially novel sites for new GABA_A_R drug development.

#### Lipoma HMGIC Fusion Partner-Like 4

Lipoma HMGIC fusion partner-like 4 (Lhfpl4, LH4; also referred to as GABA_A_R Regulatory Lhfpl4 [GARLH4]) is a four-pass transmembrane protein that was recently shown to critically regulate GABA_A_R anchoring at inhibitory synapses and thus impact the strength of fast inhibitory synaptic transmission. Indeed, both KD (Yamasaki et al., 2017) and knockout (KO) (Davenport et al., 2017; Wu et al., 2018) of LH4 resulted in decreased GABA_A_R clustering, as well as diminished GABAergic synaptic transmission and GABAergic synapse density (Davenport et al., 2017; Yamasaki et al., 2017; Wu et al., 2018). Additionally, LH4 forms a tripartite complex with GABA_A_Rs and neuroligin-2 (NL2) (Davenport et al., 2017; Yamasaki et al., 2017; Wu et al., 2018), a postsynaptic inhibitory cell adhesion molecule (Poulopoulos et al., 2009; Li et al., 2017b; Lu et al., 2017). Interestingly, the δ subunit plays an important role in the regulation of GABA_A_R assembly within the cerebellum by preventing incorporation of γ2 and LH4 (Martenson et al., 2017). Incorporation of the δ subunit prevented assembly of γ2 into GABA_A_Rs, as well as the interaction with LH4 (Martenson et al., 2017). In this manner, δ-containing GABA_A_Rs became extrasynaptically localized whereas γ2-containing GABA_A_Rs were localized at synapses via their interaction with both LH4 and NL2 (Davenport et al., 2017; Martenson et al., 2017; Yamasaki et al., 2017; Wu et al., 2018). Lastly, although LH4 did not alter sensitivity to endogenous GABA, THIP, or picrotoxin (Yamasaki et al., 2017), LH4-dependent modulation of other GABA_A_R-targeting compounds still requires further investigation.

#### Cleft Lip and Palate Transmembrane Protein 1

Abnormal trafficking of GABA_A_Rs can involve a variety of mechanisms, leading to a lack of GABA_A_R availability at the neuronal surface. Specifically, a novel transmembrane protein, cleft lip and palate transmembrane protein 1 (Clptm1) was identified as a negative regulator of GABA_A_R forward trafficking of both synaptic and extrasynaptic GABA_A_Rs through receptor confinement primarily in the ER and reduced the surface availability of GABA_A_Rs (Ge et al., 2018). Importantly, this modulation of GABA_A_R forward trafficking impacted inhibitory transmission bi-directionally; overexpression and KD of Clptm1 resulted in diminished and enhanced postsynaptic inhibitory responses, respectively. Additionally, this bi-directional effect was similarly observed in tonic currents generated from extrasynaptic GABA_A_Rs. Mechanistically, these data suggest that Clptm1 is a pan-GABA_A_R regulator and thus impacts both synaptic and tonic inhibition through restriction of receptor forward trafficking.

#### Shisa7

Members of the Shisa family of proteins are single-pass transmembrane proteins containing both cysteine and proline rich domain on the N- and C-terminus, respectively (Pei and Grishin, 2012). Specifically, Shisa6-9 are referred to as cystine knot AMPAR membrane proteins (CKAMP) (Farrow et al., 2015) due to the presence of an AMPAR interacting domain in the C-terminus (von Engelhardt, 2019). Notably, Shisa7 (CKAMP59) has emerged as an interesting member of the Shisa family in that unlike other CKAMP counterparts, Shisa7 has a direct role in GABA_A_R regulation at inhibitory synapses (Han et al., 2019). While other CKAMPs are localized at glutamatergic synapses (von Engelhardt et al., 2010; Klaassen et al., 2016; Peter et al., 2020), we observed that Shisa7 co-localizes specifically with gephyrin and GABA_A_Rs in hippocampal neurons (Han et al., 2019) and not at excitatory synapses as reported in an earlier study (Schmitz et al., 2017). Functionally, Shisa7 regulated GABA_A_R trafficking and inhibitory transmission without affecting excitatory synaptic transmission (Han et al., 2019). Strikingly, Shisa7 also modulates GABA_A_R kinetics and pharmacological properties. Indeed, in heterologous cells, Shisa7 decreased the deactivation time constants of α1β2γ2 and α2β3γ2 receptors, and conversely Shisa7 KO prolonged the decay time constant of GABAergic transmission in hippocampal neurons (Han et al., 2019). Lastly, Shisa7 increased DZ-induced potentiation of GABA_A_Rs in heterologous cells and Shisa7 KO significantly reduced DZ actions *in vivo* (Han et al., 2019). Taken together, this is the first documentation of a transmembrane auxiliary subunit unique to GABA_A_Rs that can influence receptor trafficking, kinetics, and pharmacology.

#### Targeting Transmembrane Accessory Molecules That Interact With Native GABA_A_Rs

Although 19 different GABA_A_Rs subunits have been identified, there are a multitude of different possible subunit combinations that can occur. Thus, one potential obstacle to overcome regarding the development of subtype-specific GABA_A_R-targeting drugs is achieving better therapeutic efficacy and selectivity. One strategy is to “think outside the receptor” and evaluate whether there are “druggable” targets that coexist with native GABA_A_Rs independent of the pore-forming subunits. For example, gephyrin is a well-known postsynaptic scaffolding protein that associates with GABA_A_Rs at inhibitory synapses and dysregulation of gephyrin possibly contributes to disrupted GABA_A_R signaling in disease (Tyagarajan and Fritschy, 2014). A recent preliminary study identified that artemisinin, an anti-malarial compound, could bind to gephyrin and subsequently affect GABA_A_R-mediated signaling in pancreatic cells, suggesting within this context that artemisinins could prove useful in treating diabetes (Li et al., 2017a). Additionally, crystallography studies identified that artemisinin and its derivatives bind to the GABA_A_R binding pocket in gephyrin and resulted in destabilization of gephyrin, as well as α1- and α2-containing GABA_A_Rs (Kasaragod et al., 2019). This exciting piece of evidence suggests that proteins that interact with GABA_A_Rs can be targeted and subsequently impact GABA_A_R signaling, making them ripe candidates for new drug development.

In addition to GABA_A_R-associated scaffolds and molecular adaptor proteins (Khayenko and Maric, 2019), newly identified transmembrane proteins that interact with GABA_A_Rs are additional targets that can potentially be exploited in drug development. In fact, transmembrane AMPAR regulatory proteins (TARPs), which are auxiliary subunits of AMPA receptors (Ziff, 2007; Milstein and Nicoll, 2008), are currently being evaluated for the treatment of epilepsy and pain (Maher et al., 2017). Thus, the discovery of novel GABA_A_R-associated transmembrane proteins (LH4, Clptm1, and Shisa7) as discussed above provide a potentially exciting opportunity for drug development. For example, Shisa7 can modulate GABA_A_R kinetics and pharmacology (Han et al., 2019). Thus, it will be interesting to investigate whether there are any compounds that can interact with Shisa7 and/or other transmembrane proteins, or their interfaces with GABA_A_Rs, to produce clinically relevant effects. In terms of selectivity, LH4 could potentially offer an opportunity to selectively target γ2-containing GABA_A_Rs while “sparring” δ-containing GABA_A_Rs. Collectively, these initial characterizations of transmembrane GABA_A_R regulators have provided the foundation for a new understanding of GABA_A_R-mediated mechanisms of inhibitory control (Han et al., 2020), and present new potential targets for GABA_A_R drug screening.

## Summary and Future Outlook

Characterization and investigation of the various binding sites on GABA_A_Rs have provided invaluable data for the development of pharmaceuticals that are used to treat a wide variety of neurological conditions and psychiatric disorders. However, there are still many challenges and issues to be faced with the current state of available GABA_A_R-targeting drugs. One of the biggest challenges comes from ubiquitous expression of GABA_A_R subtypes throughout the brain. This can prove to be problematic when considering off-target drug effects and unforeseen complications from drug administration due to drug binding across many GABA_A_R subtypes in different brain regions. Considering the nature of current GABA_A_R pharmaceuticals, the design of these drugs is based on previously characterized binding sites, such as the high affinity benzodiazepine binding site which exists between the α and γ subunits (Moody and Jenkins, 2018). However, there is still a lack of new and clinically relevant subunit-specific GABA_A_R-targeting drugs despite scientific success in furthering our understanding of GABA_A_R structure and function (Figure 1). The need for subtype-specific GABA_A_R-targeting drugs is not solely confined to clinical applications, but is also needed in biomedical research. The expression of specific GABA_A_Rs in unique cell populations and the lack of compounds for certain subunits, such as a commercially available δ subunit-selective antagonist, only highlights the importance for the future development of more highly selective compounds and will help address the role of specific GABA_A_Rs at the cellular, circuit, and behavioral level.

Although initial studies have characterized the role of transmembrane GABA_A_R accessory proteins within the hippocampus and cerebellum (Martenson et al., 2017; Yamasaki et al., 2017; Ge et al., 2018; Wu et al., 2018; Han et al., 2019), it has not yet been investigated whether these observations apply to other brain regions. Therefore, the possibility exists that these proteins might differentially impact GABA_A_R function within discrete brain regions which contributes to their impact on physiology and behavior, as well as the pharmacological effect of drugs. While GABA_A_Rs are expressed throughout the brain, the potential region-specific distribution of GABA_A_R-associated transmembrane proteins could enhance the selectivity for future GABA_A_R-targeting drugs. Considering this, further investigation requires cell- and circuit-specific interrogation to define how these accessory proteins function in different brain regions. Understanding how these transmembrane proteins associate with GABA_A_R and their role within defined brain structures could potentially allow for the development of drugs that target these GABA_A_R-associated transmembrane proteins directly or compounds that work synergistically with other GABA_A_R-targeting drugs to enhance their therapeutic efficacy and limit unwanted side effects.

To complement structural and binding studies, genetic studies have also proved invaluable in our understanding of GABA_A_Rs. For example, knockin mice harboring histidine-to-arginine mutations in α1- α2- α3-, or α5- GABA_A_R subunits render them insensitive to benzodiazepines (Rudolph et al., 1999; Löw et al., 2000; McKernan et al., 2000; Rudolph and Möhler, 2004) and have provided critical insight and direction toward the development of several subtype-selective ligands (Rudolph and Möhler, 2006; Rudolph and Knoflach, 2011; Richter et al., 2012; Forman and Miller, 2016; Yamaura et al., 2016; Solomon et al., 2019). However, genetic approaches also have limitations and can pose a challenge in studying specific contributions of discrete GABA_A_R subtypes, as well as modeling alterations to GABA_A_R function in disease. For example, GABA_A_R expression and subunit composition change throughout development and there are differences in GABA_A_R subtypes within various brain regions (Luscher et al., 2011), which creates difficulty in studying the role of GABA_A_R in KO models. Genetic deletion of subunits can also create complications when studying discrete subunit contributions to GABA_A_R function. For example, complete KO of the γ2 subunit results in death shortly after birth (Günther et al., 1995), preventing a functional analysis of γ2 subunit *in vivo* at later time points. Furthermore, genetic deletion of α subunits can result in compensatory effects. Indeed, deletion of the α1 subunit can promote upregulation of other α-containing subtypes in response (Sur et al., 2001; Kralic et al., 2002a, b, 2006). Additionally, deletion of the γ2 subunit can lead to compensation through replacement of synaptic GABA_A_Rs with the γ3 subunit (Kerti-Szigeti et al., 2014). Thus, the development of more selective drugs would allow for precise investigation as to the role of subtype-selective GABA_A_Rs and further elucidates the role of GABA_A_Rs in health and disease.

A major goal in GABA_A_R drug discovery has been to discover compounds that are more selective and efficacious with less side effects. Although classical benzodiazepines have been successfully used to treat a wide variety of conditions (Chen et al., 2019), their non-selective binding to essentially all γ2-containing GABA_A_Rs can account for many of their side effects (Engin et al., 2018; Chen et al., 2019). The recent emergence of several high-resolution structures of GABA_A_Rs (Laverty et al., 2017, 2019; Miller et al., 2017; Phulera et al., 2018; Zhu et al., 2018; Masiulis et al., 2019) will be important for precision-based drug design that enhances drug selectivity for discrete receptor subtypes. Excitingly, the discovery of transmembrane GABA_A_R accessory proteins will likely provide further opportunities to develop compounds that can target GABA_A_Rs in complex with other accessory proteins, but not bind and modulate GABA_A_Rs in isolation. In summary, there is a need for future interrogation of GABA_A_R pharmacology which takes into account distinct subunit compositions, discrete brain region localizations, and associated GABA_A_R proteins that better mimic native receptor complexes when designing future pharmaceuticals.

## Author Contributions

All authors listed have made a substantial, direct and intellectual contribution to the work, and approved it for publication.

## Conflict of Interest

The authors declare that the research was conducted in the absence of any commercial or financial relationships that could be construed as a potential conflict of interest.
